# Apoptosis and autophagy of muscle cell during pork postmortem aging

**DOI:** 10.5713/ab.23.0148

**Published:** 2022-10-27

**Authors:** Chunmei Li, Xialian Yin, Panpan Xue, Feng Wang, Ruilong Song, Qi Song, Jiamin Su, Haifeng Zhang

**Affiliations:** 1 College of Food Science and Engineering, Yangzhou University, Yangzhou 225009, China; 2 Key Laboratory of Chinese Cuisine Intangible Cultural Heritage Technology Inheritance, Ministry of Culture and Tourism, Yangzhou University, Yangzhou 225009, China; 3 Jiangsu Food & Pharmaceutical Science College, Huaian 223023, China; 4 Institute of Comparative Medicine, College of Veterinary Medicine, Yangzhou University, Yangzhou 225009, China; 5 Jiangsu Co-Innovation Center for Prevention and Control of Important Animal Infectious Diseases and Zoonosis, Yangzhou University, Yangzhou 225009, China

**Keywords:** Apoptosis, Autophagy, Pork

## Abstract

**Objective:**

Pork is an important source of animal protein in many countries. Subtle physiochemical changes occur during pork postmortem aging. The changes of apoptosis and autophagy in pork at 6 h to 72 h after slaughter were studied to provide evidence for pork quality.

**Methods:**

In this article, morphological changes of postmortem pork was observed through Hematoxylin-eosin staining, apoptotic nuclei were observed by TdT-mediated dUTP nick end labeling assay, protein related to apoptosis and autophagy expressions were tested by western blot and LC3 level were expressed according to immunofluorescence assay.

**Results:**

In this study, we found the occurrence of apoptosis in postmortem pork, and the process was characterized by nucleus condensation and fragmentation, formation of apoptotic bodies, increase in apoptosis-related Bax/Bcl-2 levels, and activation of caspases. Autophagy reached its peak between 24 and 48 h after slaughter, accompanied by the formation of autophagosomes on the cell membrane and expression of autophagy-related proteins beclin-1, P62, LC3-I, LC3-II, and ATG5.

**Conclusion:**

Obvious apoptosis was observed at 12 h and autophagy reached its peak at 48 h. The present work provides the evidence for the occurrence of apoptosis and autophagy during postmortem aging of pork. In conclusion, the apoptosis and autophagy of muscle cells discovered in this study have important implications for pork in the meat industry.

## INTRODUCTION

Pork is one of the important sources of animal protein in many countries with important nutritional and economic significance [1]. There is a growing demand for high-quality pork with desirable color, tenderness and flavor. Studies suggested that complex physiological and biochemical reactions during postmortem aging may play an important role in meat quality [2]. Transportation stress, storage temperature, slaughter methods, and nitric oxide application can affect glycolysis. Nitric oxide could promote the activation of AMP-activated protein kinase, thus accelerating glycolysis [3]. Pork refrigeration conditions and aging time have an impact on the tenderness and flavor of pork, which will directly affect consumers’ desire to buy. In addition, changes in muscle energy metabolism and physiology processes, such as apoptosis and autophagy during postmortem aging play a key role in the regulation of meat quality [4]. However, the mechanism and regulation of such changes are still unclear.

After the pigs are slaughtered, the muscles are in a tense state, irreversible contraction occurs in the muscle fibers and lactic acid is produced in the carcass, When the meat reaches a certain state, the internal protein degrades into molecular peptides and amino acids, and the taste and tenderness of the muscles improves, which is the maturation process of meat. Apoptosis and autophagy affect the maturation process of meat. Apoptosis refers to autonomous and highly organized programmed cell death and plays a role in homeostasis [5]. It can be induced by intracellular mitochondrial signals or extracellular death receptors [6]. Hypoxia or the ischemia of muscle cells may increase the rate of anaerobic metabolism, decrease cellular ATP and pH levels, and induce calcium release. These factors are involved in endogenous mitochondrial or endoplasmic reticulum-related cell death signaling pathways and induce muscle cell apoptosis during postmortem aging [7,8]. In the process of pork sale, consumers are particularly concerned about the of pork. Postmortem tenderization improves meat quality and tenderness is one of the most important factors for consumers to evaluate the quality of pork [9]. Apoptosis plays a major role in the postmortem tenderization of meat after death [10]. Caspases act as the key effector molecules of apoptosis. Changes in caspase activity could affect muscle quality during pork aging [11]. The Caspases family such as Caspase-3 and Caspase-6 are involved in the hydrolysis of muscle fibrin after slaughter, and their activity is positively correlated with the tenderness of meat. Evans et al [12] proposed that the occurrence of cell apoptosis triggered an imbalance between the rate of muscle protein synthesis and degradation. Therefore, the molecular regulation mechanism of apoptosis is related to the quality of pork after slaughter.

In apoptosis, characteristic morphological changes such as cell shrinkage, chromatin condensation, apoptotic body formation can be observed. Autophagy is also involved in the maturation process of meat after slaughter [13]. Studies had proved that autophagy affects the activity of caspases enzyme and indirectly affects the tenderness of meat. Autophagy can also activate the enzyme activity of apoptosis and regulate the mechanism of apoptosis [14]. Therefore, the study of postmortem autophagy is of significance to the quality of pork, but research on autophagy is relatively scarce. Besides, autophagy is initiated in cells in response to nutrient deficiency, metabolic imbalance, and oxidative stress [15]. During postmortem aging, oxidative stress produced by reactive oxygen species is related to the occurrence of skeletal muscle cell apoptosis, autophagy, and necrosis [16,17]. At the end of autophagy, morphological changes, including an increase in number, and enlargement, gray-white components inside autophagosomes, and minor nuclear pyknosis can be observed [18]. Regarding the autophagy-related genes, as the homologous gene of Atg6, beclin-1 is the first specific gene that mediates autophagy in mammalian cells. The levels of apoptosis and autophagy in pork tissues change with time even in low temperature environment, so we evaluated the quality of pork by measuring the changes of apoptosis and autophagy in different shelf-life periods. Our present study investigated the occurrence and mechanism of muscle cell apoptosis and autophagy during postmortem pork aging and further explored the mechanism of postmortem pork aging, so as to provide theoretical reference for the development of pork in food industry.

## MATERIALS AND METHODS

### Experimental design

The 6-month-old crossbred boars with live weight of 100 ±10 kg were acquired from Xiangtai Food Co., LTD (Yangzhou, Jiangsu, China). The implementation of the slaughter process was based on the Operating Procedures of Livestock and Poultry Slaughtering-Pig (GB/T 17236–2019). Briefly, crossbred boars without feeding for 12 hours before slaughter, the *longissimus thoracis* muscles were taken and refrigerated at 0°C to 4°C for maturation. The related indexes were detected at 6, 12, 24, 48, and 72 h of postmortem maturation. Part of meat samples were rapidly placed in liquid nitrogen and then frozen at −80°C for biochemical analysis while other samples were fixed with 1% paraformaldehyde for morphological observation. Animal studies were approved by the Jiangsu Administrative Committee for Laboratory Animals, the approval number of IACUC is A12070201.

### Hematoxylin-eosin staining

The 2 cm of fresh intestine was flushed and fixed in 4% paraformaldehyde solution. The intestines were buried in paraffin and 4 to 5 μm slices were prepared. The slices placed on a slide were stained by hematoxylin-eosin (HE) for immunohistochemical analysis.

Briefly, the slices were immersed in pure xylene solution for 20 min until the paraffin was completely dissolved. Then the slices were placed in the mixed solution of xylene and anhydrous ethanol (v/v, 3/4), xylene and anhydrous ethanol (v/v, 1/2), and xylene and anhydrous ethanol (v/v, 1/4), respectively. After each reaction for 10 min, the slices were rehydrated in 100%, 95%, 90%, 80%, 70%, 60%, and 50% ethanol solutions with each reaction for 10 min. Afterwards, the slices were stained with hematoxylin for 4 min, and then washed with running water. Hydrochloric acid and alcohol solution (v/v, 1/100) was added for reaction and subsequently washed with running water. The obtained sections were dyed in eosin solution for 90 s and washed with ultra-pure water. Then 25%, 50%, 75%, and 100% xylene solution was applied in the slices. Each reaction proceeded for 10 min. Finally, the slices were mounted by neutral balsam (Beyotime, Shanghai, China) and then observed under a microscope (BX53; Olympus, Tokyo, Japan) to analyze the morphology of the nuclei. Images were acquired at 400× magnification.

### TdT-mediated dUTP nick end labeling assay

The TdT-mediated dUTP nick end labeling (TUNEL) assay was performed for the analysis of apoptosis. The frozen sections were rinsed twice with a phosphate buffer solution (PBS) buffer and stained according to the manufacturer’s instructions (Servicebio, Hubei, China). The tissue slices were mounted with glycerol and observed under a super-resolution laser confocal microscope (TCS SP8 STED; Leica, Wetzla, Germany). Each sample was photographed in at least three different fields at 400× magnification. Apoptotic and total nuclei were recognized according to the red fluorescence of TUNEL and blue fluorescence of 4′,6-diamidino-2-phenylindole (DAPI), respectively.

### Western blot

The pork samples at different postmortem aging times were washed with PBS and lysed with lysis buffer (Applygen, Beijing, China) including protease and phosphatase inhibitor cocktail (New Cell Molecular Biotech, Suzhou, China) for 30 min. The mixtures were centrifuged at 12,000×g for 10 min at 4°C. The protein concentration of supernatant was determined using bovine serum albumin (BSA) kit (Thermo Fisher Scientific, Waltham, MA, USA) and adjusted to the same concentration. To prepare gel samples, protein samples were mixed with loading buffer (100 mM Tris-HCl, 5 mM ethylene diamine tetraacetic acid, 20% glycerol, 4% sodium dodecyl sulfate (SDS), 1% β-mercaptoethanol, 0.01% bromophenol blue) and then boiled at 95°C for 5 min. Subsequently, protein samples were separated by polyacrylamide gel electrophoresis. After electrophoresis, the proteins were transferred to polyvinylidene difluoride membrane (Bio-Rad Laboratories, Hercules, CA, USA). Subsequently, 5% BSA was used for the blocking of proteins for 2 h. The membranes were then incubated with primary antibodies overnight at 4°C. After washing three times by Tris buffered saline with tween-20 (TBST) for 5 min each, the membranes were incubated with secondary antibody at room temperature for 1 h. After washing again, chemiluminescence reagents (New Cell Molecular Biotech, Suzhou, China) were used to develop the protein bands. The bands were imaged by the chemiluminescence imaging analysis system (ProteinSimple, Silicon Valley, CA, USA) and then quantified using NIH Image-J software.

The primary antibodies include BCL2-associated X (Bax, ab104156), B-cell lymphoma-2, (Bcl-2, ab117115), glyceraldehyde-3-phosphate dehydrogenase (GAPDH, ab9484), Beclin-1 (ab231341), sequestosome-1 (P62, ab233207), and autophagy-related 5 (ATG5, ab228668) were acquired from abcam (Cambridge, Britain), Caspase-3 (#14220), Caspase-8 (#4790), microtuble-associated protein light chain 3-I/II (LC3-I/II, #12741) were acquired from Cell Signaling Technology (Boston, MA, USA). The secondary antibodies include Anti-rabbit immunoglobulin G (IgG), horseradish peroxidase-linked (HRP-linked, Antibody (#7074) and Anti-mouse IgG, HRP-linked Antibody (#7076) sourced from Cell Signaling Technology (USA).

### Immunofluorescence assay

The optimal cutting temperature compound adhesive coated part of the tissue was placed into liquid nitrogen, then removed and placed on the frozen microtome (CM1950; Leica, Germany). The tissues were cut to 8 μm and adsorbed to an adhesive slide, allowed to stand at room temperature for 5 min. Paraformaldehyde solution (4%) was added to the drops and fixed at room temperature for 10 to 15 min, then dried in a fume cupboard, and stored in a section box at −20°C.

The prepared frozen sections were washed with PBS and blocked in 5% bovine serum at room temperature for 1.5 h and incubated with an LC3B (-II) rabbit antibody (ab229327; abcam, China) overnight at 4°C. Fluorescent secondary antibodies (ab150080; abcam, China) were applied to the tissue sections in the dark for incubation at 37°C for 2 h. After incubation, the slices were stained with DAPI for 10 min. After fixation, the sections were observed under an ultra-high resolution laser confocal microscope (TCS SP8 STED; Leica, Germany).

### Statistical analysis

All tests were carried out in triplicate. The results were represented as mean±standard error and analyzed with GraphPad Prism 8.0 software. In the statistical model, storage time was the fixed variable, and muscle was considered as random variable. One-way analysis of variance was performed using SPSS 19.0 software. A p-value of <0.05 was considered statistically significant.

## RESULTS AND DISCUSSION

### Morphological changes in postmortem pork

**Figure 1:**
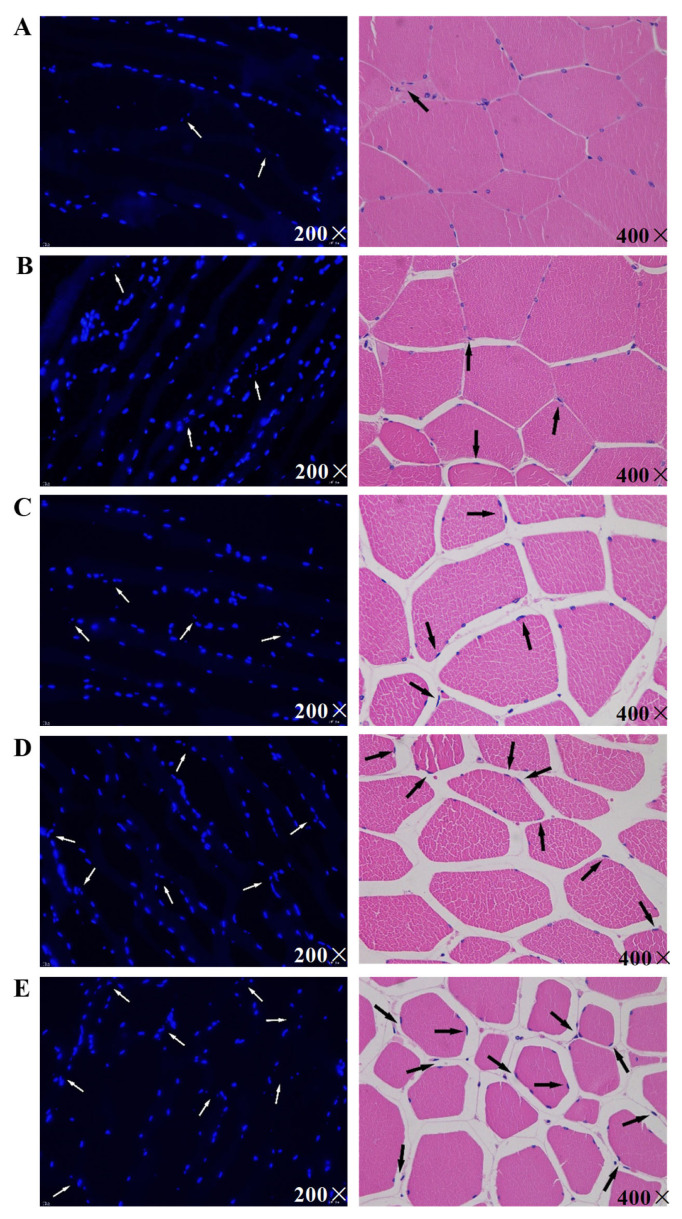
Morphological changes during pork postmortem aging through hematoxylin-eosin (HE) staining. Morphological changes were marked with arrowheads.

Observing the changes of nuclear morphology is an effective method for detecting cell apoptosis. Generally, when cells undergo apoptosis, cell shrinkage, chromatin condensation, and apoptotic bodies formation are detected [19]. In this study, morphological changes of postmortem pork were observed through DAPI staining (Figure 1A–1E) and HE staining (Figure 1F–1J).

The nuclei of skeletal muscle cells were stained blue by DAPI. The nuclei at 6 h postmortem was basically intact and uniform in size. The level of nuclei pyknosis and irregularity increased over time. At 12 h postmortem, the nuclear staining became brighter, and particles increased. The white arrows in Figure 1A–1E point to typical apoptotic nuclei, which gradually disintegrated and ruptured to form multiple apoptotic bodies or fragments.

Through HE staining, the nucleic structures of pork muscle cells at 6 h postmortem showed retained integrity, clear outlines, and uniform nuclear and cytoplasm distributions. However, after 6 h postmortem, the intercellular space gradually increased with the extended postmortem aging time. As indicated by the black arrows in Figure 1F–1J, at 12, 24, 48, and 72 h postmortem, some parts of the nucleus and cytoplasm gradually migrated to edge, half-moon-shaped apoptotic bodies formed, and the nuclei shrunk. These phenomena implied that apoptosis occurred in postmortem pork muscle cells. Apoptosis in pork occurred 6 to 12 h after slaughter. These results are consistent with the results of Dirks and Leeuwenburgh [20] and Ouali et al [21] who indicated that apoptosis might occur in the early stage of muscle aging.

### TdT-mediated dUTP nick end labeling analysis

**Figure 2:**
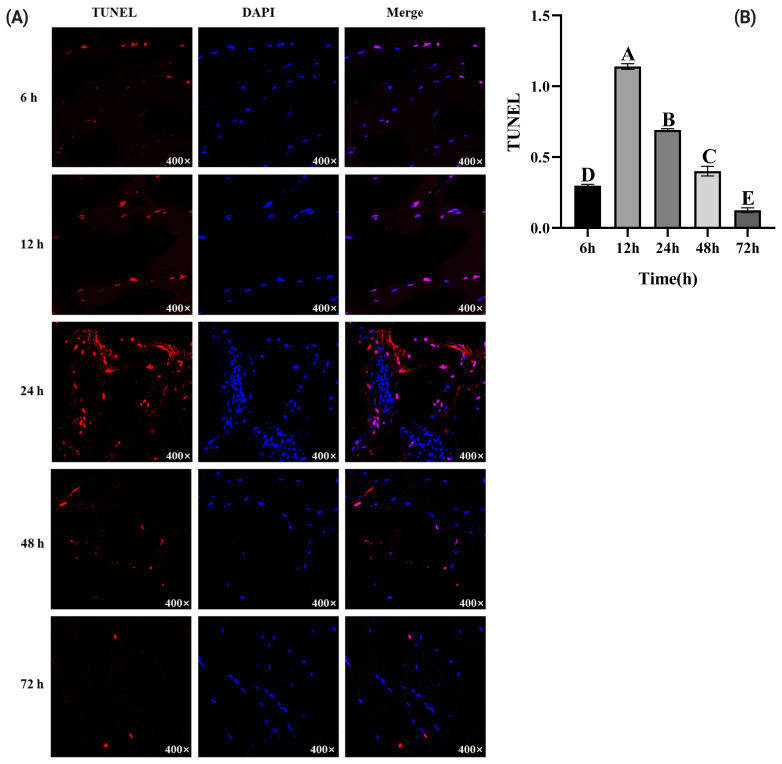
Changes of apoptosis levels during pork postmortem aging through the TdT-mediated dUTP nick end labeling (TUNEL). (A) Apoptotic and total nuclei were recognized by red fluorescence of TUNEL and blue fluorescence of DAPI, respectively. (B) The ratio of the red/blue fluorescence was used to calculate the apoptosis rate. DAPI, 4′,6-diamidino-2-phenylindole. ^A–E^ Different letters indicate significant differences (p<0.05, n = 3).

When apoptosis occurs, chromosomal DNA is initially cleaved by endonucleases, and then two strands of DNA break to form 3′-OH ends, thereby forming nucleosomal DNA multimers [22]. Through TUNEL, fluorescently labeled deoxynucleotides can covalently link to 3′-OH, which has the advantages of high sensitivity and fast response, and thus being widely applied in detecting cell apoptosis [23]. Combined with DAPI staining, TUNEL accurately reflects DNA breakage and staining location in the nuclei, and the contrast between positive cells and negative cells is clearly identified. Blue-red staining in the nuclei indicated apoptotic nuclei, and normal nuclei were only stained blue by DAPI (Figure 2A). At 6 h postmortem, apoptotic nuclei appeared, and the number of apoptotic nuclei significantly increased (p<0.05). At 12 h postmortem, the number of apoptotic nuclei rapidly increased and reached its maximum. Subsequently, the number of apoptotic nuclei gradually decreased (Figure 2B). However, the results are not consistent with the apoptosis in yak meat, which showed the highest apoptotic state at 6 h after slaughter [20]. The differences between these results may attribute to the differences in the types of muscle fibers in yak meat and pork, and differences in mitochondria, cytochrome-C content, and energy. Pistilli et al [24] indicated that fiber type could affect early energy metabolism in postmortem muscles, and energy metabolism disorders are closely related to apoptosis [25].

### Expression of apoptosis-related proteins

**Figure 3:**
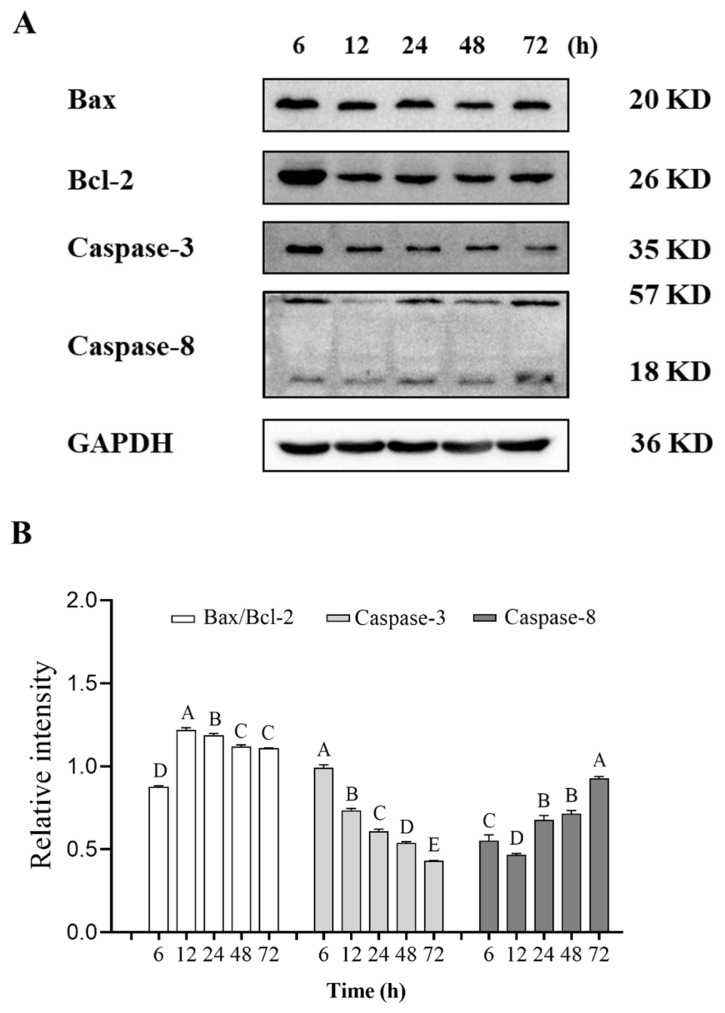
Expression of apoptosis-related proteins including Bax, Bcl-2, caspase-3, and caspase-8 in postmortem pork during aging. (A) Representative western blot graphs of Bax, Bcl-2, caspase-3, and caspase-8. (B) The relative contents changes of Bax, Bcl-2, caspase-3, and caspase-8. ^A–E^ Different letters indicate significant differences (p<0.05, n = 3).

Apoptosis is closely related to the Bcl-2 family, which can regulate apoptotic processes by maintaining the balance between the level of Bcl-2, the apoptosis inhibitor, and Bax, the apoptosis promoter [26]. The relative content of Bax/Bcl-2 in pork significantly increased at 6 h and 12 h postmortem and reached the maximum value at 12 h postmortem (Figure 3). This result is consistent with that of Guo et al [27] who reported that the Bax/Bcl-2 level decreased gradually with the increasing of aging time. Manna et al. proposed that the ratio of Bax to Bcl-2 in fish was calculated and used in determining whether cells underwent apoptosis [28]. In this experiment, changes in Bax/Bcl-2 level during pork aging indicated that the apoptosis of pork muscle cells occurred after slaughter, and the highest rate of the process was observed at 12 h postmortem.

Caspase-3, one of the essential members of the caspase family, is considered a key effector molecule and an important executive protein in apoptosis [29]. Its activation marks the beginning of irreversible apoptosis. Caspase-3 was produced in pork muscle cells after slaughter, and its expression obviously decreased at 6 h postmortem (Figure 3). During postmortem aging of pork, the apoptosis signal was amplified after the release of mitochondrial cytochrome C and the formation of apoptotic bodies. Then a caspase cascade reaction was initiated, with caspase-3 being activated and then acting on the degradation of downstream proteins [30]. Zhang et al [31] reported that caspase-3 was degraded into 23 and 17 kDa fragments during postmortem beef aging. This process was considered in the present study.

Caspase-8 is involved in the initiation of apoptosis and activated caspase-8 can participate in the activation of the downstream effector caspase, such as caspase-3 and caspase-7. In postmortem pork, caspase-8 was cleaved into 18 kDa activated fragments, and the relative content generally showed a trend of first decreasing (6 to 12 h postmortem) and then increasing (12 to 72 h postmortem). Similarly, an 18 kDa fragment of caspase-8 was found in the comparative research of different pork skeletal muscles [32]. The result suggests that caspase-8 is activated under hypoxia in the early postmortem stage of pork.

### Changes of LC3 level during pork postmortem aging

**Figure 4:**
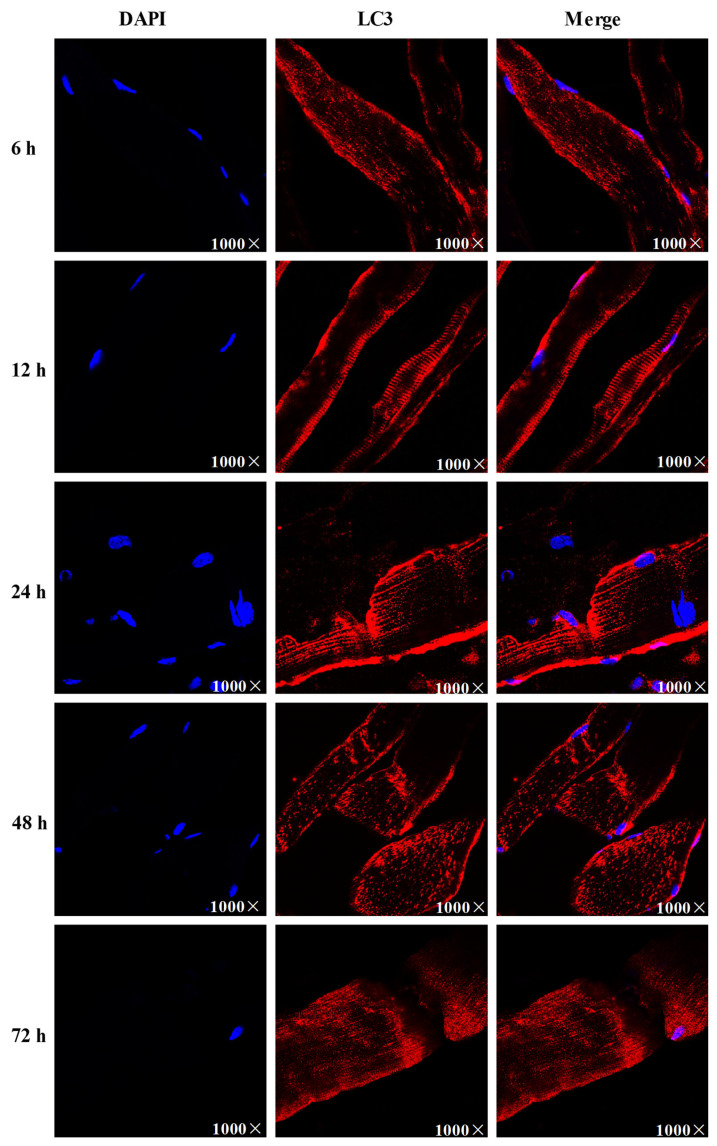
LC3 (red) level and DAPI (blue) changes during pork postmortem aging. LC3, microtuble-associated protein light chain 3; DAPI, 4′,6-diamidino-2-phenylindole

LC3 is a marker for autophagosome membranes, and red fluorescence indicates the formation of autophagosomes. The results showed that at 6 and 24 h postmortem, red fluorescence appeared as puncta in pork muscle cells, and the fluorescence intensity gradually increased, suggesting that autophagosomes were formed on the cell membrane at early postmortem, and the level of autophagy was significant increased (Figure 4). However, the amount of red fluorescence spots decreased at 48 and 72 h, and then red fluorescence distributed diffusely, indicating that the level of autophagy decreased over time.

### Expression of autophagy-related proteins in postmortem pork

**Figure 5:**
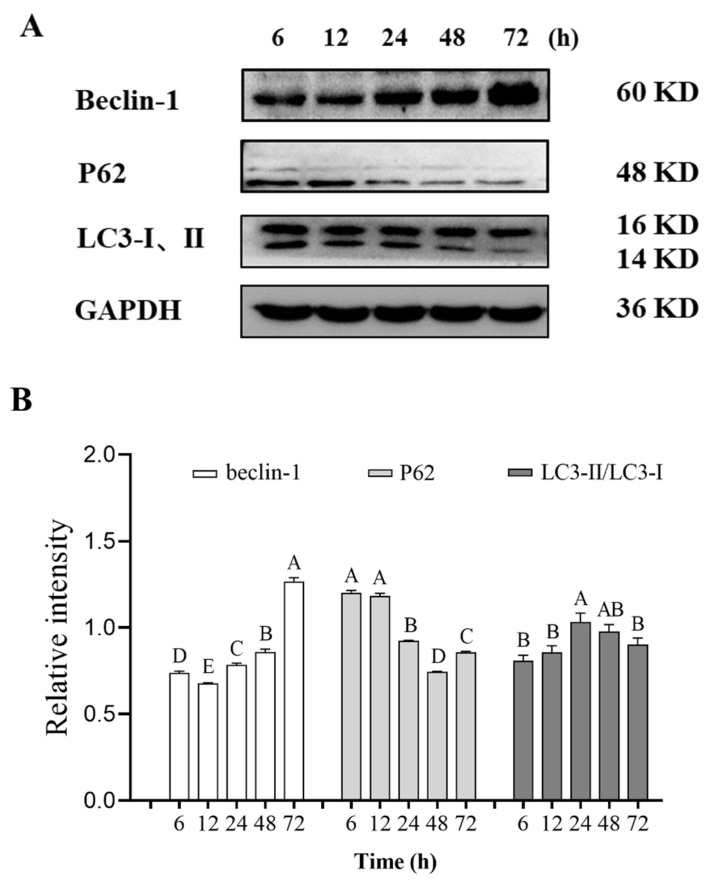
Expression of autophagy-related proteins in postmortem pork during aging. (A) Western blot graphs of Beclin-1, P62, and LC3-II. (B) The relative contents of Beclin-1, P62, and LC3-II, which was calculated by Image. ^A–E^ Different letters indicate significant differences (p<0.05, n = 3).

Autophagy is a multi-step process involving multiple proteins. Beclin-1 is one of the important proteins in the initial stage of autophagy, which plays a role in autophagy regulation and participates in the formation of autophagosomes [33]. At 12 and 24 h postmortem, the relative content of beclin-1 decreased and then increased compared with 6 h postmortem. When aging time was extended from 12 to 72 h after slaughter, the relative content of beclin-1 in pork showed an overall upward trend (Figure 5). These results indicate that the occurrence of autophagy proceeded gradually and persisted in the entire postmortem aging period.

Another important protein is the autophagy substrate P62 protein, which is a multi-domain ubiquitin-binding protein encoded by the recombinant sequestosome 1 *(SQSTM1*) gene and plays an important role in the ubiquitin-proteasome system and autophagy [34]. In autophagy, the expression of P62 gradually decreased with the enhancement of autophagy, which was usually negatively correlated with the activity of autophagy. The relative expression of P62 in this experiment showed a significant downward trend at 12 and 48 h postmortem, indicating that autophagy was enhanced during this period (Figure 5).

LC3-II is a key protein in autophagy and localized on the surface of pre-autophagic vacuoles and autophagic vesicles, which is considered a specific marker of autophagic activity [35]. When autophagy occurs, cytoplasmic LC3 (LC3-I) enzymatically degrades small segments of polypeptides into the autophagosome membrane LC3-II. The LC3-II/I ratio is usually estimated according to the level of phagocytosis. Within 72 h after slaughter, the relative content of LC3-II/LC3-I in pork increased first and then decreased. This result is consistent with the expression of LC3-II/LC3-I in Figure 5. The early increase may be inseparable from the mediating effect of beclin-1 [36]. Moreover, during autophagosome membrane formation, prophase LC3 may be cleaved into LC3-I, which binds to phosphatidylethanolamine to form LC3-II and thus prolonging autophagy [37]. Upon the formation of autophagosomes, LC3-II gradually aggregated and localized on the autophagosome membrane. When autophagosomes fuse with lysosomes and are degraded, LC3-II can be re-lipidated to LC3-I, which may be involved in the formation of autophagy [38].

**Figure 6:**
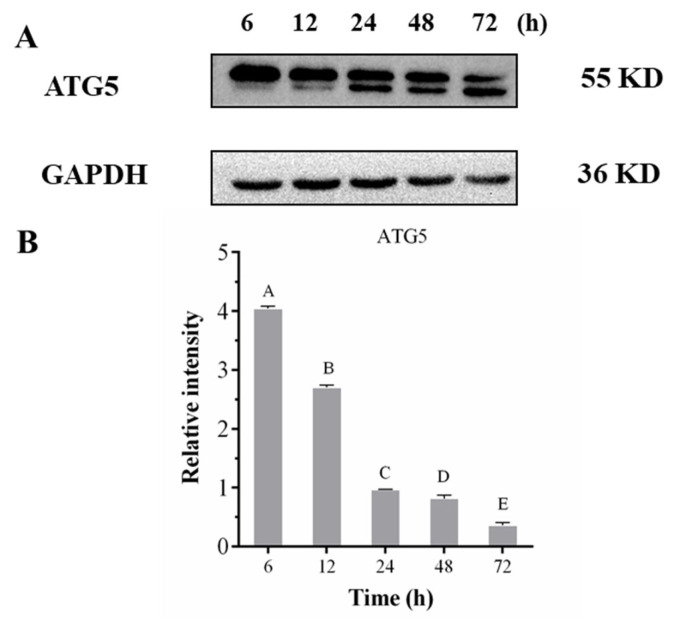
Expression of Atg5 during the postmortem pork aging. (A) Western blot graph of Atg5. (B) The relative content changes of Atg5. It was calculated by Image. ^A–E^ Different letters indicate significant differences (p<0.05, n = 3).

ATG5 is an essential protein for autophagosome formation. Complexes conjugated with ATG12, which can determine the bending direction of pre-autophagosome membrane, promote the extension and expansion of the membrane, and further produce esterification and activate autophagy. Besides, it plays an essential role in autophagy extension [39]. Western blot results showed that ATG5 exhibited two bands (Figure 6). One band was attributed to the endogenous level of the ATG5 total protein, and another was attributed to the conjugation of ATG5 ATG12. The relative expression of total protein showed a significant downward trend (p< 0.05) during postmortem aging, and the level of ATG5 that conjugated with ATG12 showed an obvious upward trend. The results demonstrate that autophagy occurred in postmortem pork.

## CONCLUSION

The identification of pork quality is helpful to the development of the pork meat industry. Apoptosis and autophagy of pork are directly related to pork quality. A series of complex physiological and biochemical reactions occur in the muscle after death, mainly including glycolysis, muscle contraction, apoptosis, protein degradation, etc. These reactions largely determine the formation of meat quality. The improvement of pork quality can be achieved by influencing the biochemical reaction during postmortem aging [40,41]. The present work provides evidence for the occurrence of apoptosis and autophagy during postmortem aging of pork. It was characterized by the gradual disintegration and rupturing of the nucleus, formation of multiple apoptotic bodies, as well as the activation of apoptosis-related proteins. The number of apoptotic nuclei and the expression of related proteins show that apoptosis is intensive at 12 h postmortem. Subsequently, autophagy reaches its peak at 48 h after slaughter, accompanied by the gradual formation of autophagosomes on the cell membrane, and changes in the expression levels of autophagy-related proteins (beclin-1, P62, LC3-I, LC3-II, and ATG5). Previous studies have found that oxidative stress occurred, and reactive oxygen produced after slaughter, which leads to mitochondrial lipid peroxidation, changes in mitochondrial permeability and ATP levels, and caspase family activation, thus affecting the tenderness of meat. At the same time, the BCL family is also involved in muscle postmortem apoptosis, which is consistent with our studies [42]. Zhang et al [43] showed that cell membrane permeability increased, calcium ion level increased firstly and then decreased, pork muscle exhibited apoptosis in the early stage of postmortem aging and tenderness in the late stage of postmortem aging, and protein phosphorylation regulated cells apoptosis by regulating the combination of ATP and calcium ion. The apoptosis level was higher at 12 h and autophagy level was higher at 24 h. Our research is beneficial to the application of pork in practical industry and provides theoretical reference for the change of pork quality in different periods.
